# Mathematical model for the distribution of major depressive episode durations

**DOI:** 10.1186/1756-0500-7-636

**Published:** 2014-09-12

**Authors:** Shinichiro Tomitaka, Toshiaki A Furukawa

**Affiliations:** Department of Mental Health, Panasonic Health Center, Landic building 3F, Nishishinbashi 3-8-3, Minato-ku, Tokyo 105-0003 Japan; Department of Health Promotion and Human Behavior, Kyoto University Graduate School of Medicine/School of Public Health, Yoshida Konoe-cho, Sakyo-ku, Kyoto 606-8501 Japan

**Keywords:** Depression, Log-normal distribution, Power law distribution, Mathematical model

## Abstract

**Background:**

The duration of major depressive episodes varies widely, ranging from one month to more than several years. Despite the accumulation of knowledge regarding the course of major depressive episodes, no mathematical model has been developed to describe the durations of major depressive episodes. We evaluated which mathematical model is fitted to describe the distribution of the durations of major depressive episodes using data from the Group for Longitudinal Affective Disorder Study (GLADS), a prospective study conducted in Japan.

**Results:**

The distribution of the cumulative probability of major depressive disorder duration plotted on a double-logarithmic scale exhibited an approximately linear form. A log-normal distribution fit the distribution of major depressive episodes better than an exponential distribution or a Weibull distribution.

**Conclusions:**

In this study, we evaluated which mathematical model fit the distribution of major depressive episode durations using data from GLADS. The results showed that a log-normal model and a power law model may fit the distribution of major depressive episode durations.

## Background

How long does an episode of unipolar major depression last? Studies available to date suggest that the median time to recovery of a major depressive episode is 3–12 months in cohort studies [1, 2], with rates of chronicity (a duration of 24 months or more) of between 10% and 30% [3, 4]. These considerable rates of chronicity suggest a long-tailed distribution, which can be partly explained by the fact that longer durations of depression are associated with lower rates of recovery [5].

Despite the accumulation of knowledge regarding major depressive episodes, there seems to be no overarching theory to explain the distribution of major depressive episode durations. The difficulty of developing a model describing the distribution of major depressive episode durations lies in the diversity of the results of each study. The indexes of distribution, such as the median time to recovery and the rates of chronicity, differ widely among previous studies. Furthermore, since a portion of depressive episodes exceeds the given period of the cohort studies, the average or variance of the distribution of major depressive episodes is difficult to estimate.

A survival analysis is used to estimate the distributions of major depressive episode durations. In general, survival curves are governed by non-normal distribution models, such as an exponential distribution, Weibull distribution, or log-normal distribution [6]. However, which model best fits the distribution of major depressive episode durations has received little attention.

Whereas the indexes of the distributions of major depressive episodes differ widely among previous studies, the survival curves of major depressive episode durations appear to exhibit a similar shape characterized by a positively skewed distribution with a long tail [1–5]. The similarity of the survival curve shapes suggests that major depressive episode durations may follow a specific mathematical distribution.

We evaluated whether a mathematical model can be used to describe the distribution of major depressive episode durations using data from the Group for Longitudinal Affective Disorder Study (GLADS), a prospective study conducted in Japan [3].

## Methods

### Analysis data

We used data from GLADS, which was a 2-year multi-center prospective follow-up study of patients with a mood disorder: the patients were selected so as to be representative of untreated, first-visit patients at 23 psychiatric settings from all over Japan. The details of GLADS have been published elsewhere [3]. The GLADS study was approved by the ethics committees of Nagoya City University School of Medicine and Tokyo Women’s Medical University Hospital and present study has been permitted to use the data. Written informed consent was obtained from all the subjects. The cohort consisted of 90 subjects who were diagnosed as suffering from unipolar major depressive disorder, not superimposed on dysthymic disorder, according to the DSM-IV. Each patient in the cohort was treated with, on average, 60 mg of imipramine or equivalent per day upon entry and 85 mg per day at 1 month. Recovery from a major depressive episode was defined in accordance with the definition of the US National Institute for Mental Health (NIMH) as 2 consecutive months with no more than one or two symptoms of mild depression [5]. Patients who recovered within a few days after the commencement of treatment were regarded as having recovered at 0.5 months.

### Statistics

The cumulative probability of the durations of major depressive episodes was estimated using the Kaplan Meier method. A set of the estimated cumulative probabilities of the major depressive episode durations was plotted on probability paper for an exponential distribution, a Weibull distribution, and a log-normal distribution. A fitting curve was calculated using the maximum likelihood estimation method.

Since the survival curve showed an approximately linear pattern when plotted using a double-logarithmic scale, the power law trend line was calculated using the least squares method. JMP version 11 for Windows was used for all the statistical analyses.

## Results

**Figure 1:**
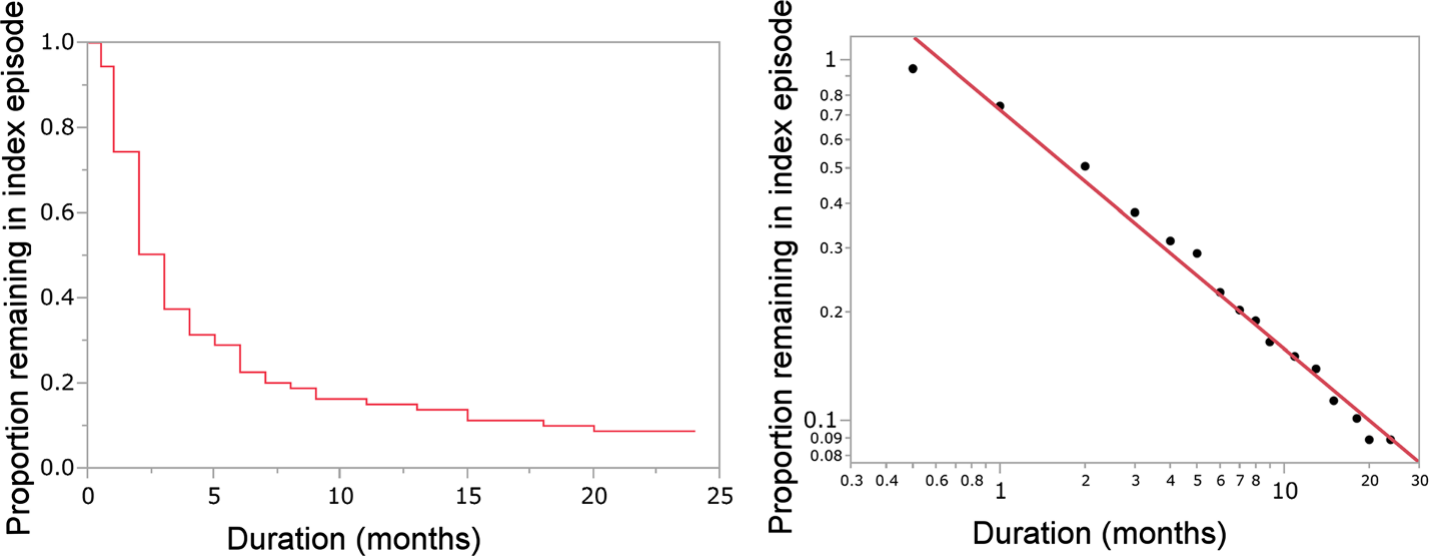
**Left: survival curve of a cohort (n = 90) with previously untreated DSM-IV major depressive disorder remaining in the index episode after the start of treatment.** Right: another scattergram of the estimated cumulative probability plotted on double-logarithmic scales.

Figure 1 shows the cumulative probability of the previously untreated subjects with DSM-IV major depressive disorder (n = 90) remaining in the index episode after treatment commencement. The median time to recovery from the index episode after treatment was 3 months: 26% of the cohort reached an asymptomatic or minimally symptomatic status by 1 month, 63% by 3 months, 85% by 12 months, and 91% by 24 months.

A set of estimated cumulative probabilities of major depressive episode durations plotted on double-logarithmic scales (Figure 1: Left) showed an approximately linear relation. The trend line was calculated using a power law model and the observed data (logy = −0.209731 - 0.7090606*logx).

**Figure 2:**
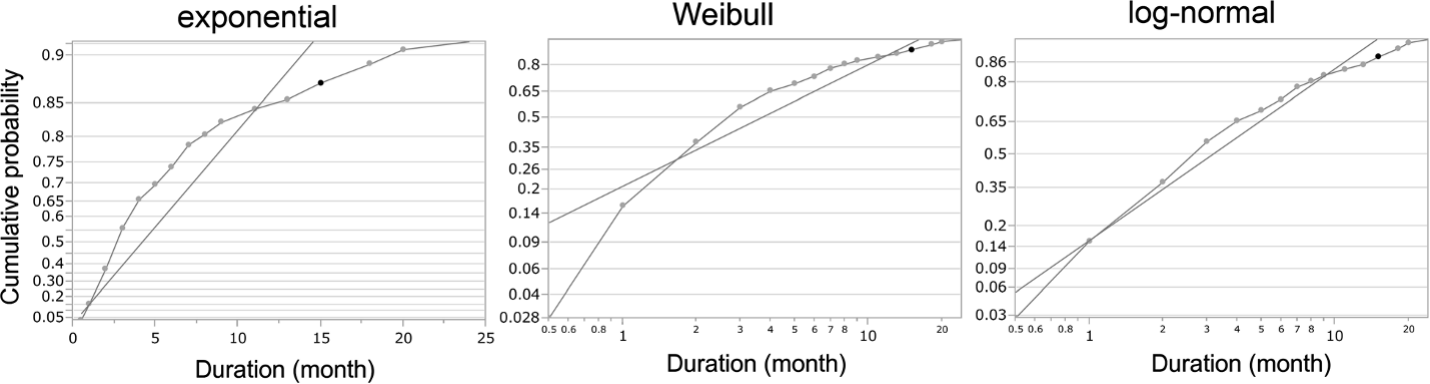
**Left: probability paper for exponential distribution.** The fitting curve parameter is θ = 5.99. Center: probability paper for Weibull distribution. The fitting curve parameters are α = 5.61 and β = 0.85. Right: probability paper for log-normal distribution. The fitting curve parameters are μ = 1.15 and σ = 1.14.

To evaluate the model of the distribution, a set of estimated cumulative probabilities of major depressive episode durations was plotted on probability paper for an exponential distribution, a Weibull distribution, and a log-normal distribution (Figure 2). The markers on the probability paper tend to follow a straight line when the distributional fit is suitable for the data. Figure 2 shows that the log-normal plot was the closest to a straight line among the three distributions, indicating that a log-normal distribution had the best fit with the observed data.

## Discussion

The goal of this study was to assess which model best fit the survival curve for major depressive episode durations. Our findings showed that a log-normal distribution fit the distribution of major depressive episode durations better than that of an exponential distribution or a Weibull distribution.

A log-normal distribution is a probability distribution for a random variable for which the logarithmic values are normally distributed. Empirically, a log-normal model is accepted as a survival curve model [7]. In fact, a variety of medical examples fit a log-normal distribution. For example, the latent periods (time from infection to first symptoms) of infectious diseases [8, 9], the survival time after a diagnosis of cancer [10, 11], and the age of onset of diseases and the duration of elderly disabilities [12] have been shown to fit a log-normal distribution.

The underlying process of a log-normal distribution is regarded as a “multiplicative process,” whereas a normal distribution is generated by an “additive process” [13]. When a process requires the completion of sequential subprocesses, the probability of the success or failure of the primary process is considered to show a log-normal distribution because of the central limit theorem [14]. Inversely, if the survival curves of a disease follow a log-normal distribution, disease recovery or progression is thought to occur according to a multiplicative process.

The finding that the distribution of major depressive episode durations fitted a log-normal distribution best suggests that recovery from a major depressive disorder may occur according to a multiplicative process. Environments, genotypes, treatments, and biological systems may be involved in the recovery of major depressive episodes on a multiplicative, not additive, basis.

Interestingly, the distribution of the cumulative probabilities of major depressive disorder durations plotted on a double-logarithmic scale exhibited an approximately linear form, indicating that the duration of depressive episodes seems to follow a power law distribution. However, as a survival curve model, the use of a power law distribution is obscure.

It is not surprising that both a log-normal distribution and a power law distribution arose together as possible distributions. Log-normal distributions and power law distributions are connected both theoretically and empirically [15]. Whereas the multiplicative model is used to generate a log-normal distribution, only a small change from a log-normal generative process yields a generative process with a power law distribution. As long as there is a bounded minimum that acts as a lower reflective barrier to the multiplicative model, a power law distribution, rather than a log-normal distribution, will be generated [16]. As a matter of fact, arguments over whether a log-normal or a power law distribution is a better fit for some empirically observed distributions have been repeated across many fields, including financial models [17], biology [18], chemistry [19], ecology [20], and information theory [21].

A wide range of data is necessary to determine whether a log-normal or a power law distribution is a better fit for empirically observed distributions. Whereas a log normal distribution will appear as a nearly straight line, similar to a power law distribution, when plotted using a double-logarithmic scale for a large portion of the body of the distribution, the log-normal distribution will appear to have a curved nature in the tail region [16]. However, data for major depressive episode durations of more than two years were not available in our study. Because of this limitation, we were unable to determine whether a log-normal or a power law distribution was a better fit to the observed distribution.

This study has some limitations. First, the sample size (N = 90) of the data from the GLADS is relatively small for analysis. Further studies with large sample size are needed to establish our findings. Second, this analysis did not examine any potential factors, such as type or dose of antidepressant, comorbidity and personality, which influence the major depressive episode durations. Finally, although a log-normal distribution had the best fit with the observed data, we did not perform authentic curve fitting.

Mathematically, there must be a necessary condition for a power law to arise during recovery from major depression. The instantaneous change in the cumulative probability of a cohort remaining in the index episode was defined as , where N is the number of patients remaining in the index episode and  is the derivative of the number of patients remaining in the index episode. When  is inversely proportional to the duration of the depressive episodes, the following formula can be produced:
1

where k is a constant and N, t, k > 0.

The following power law equation can then be obtained by solving the differential equation (1):
2

where A is a constant > 0.

Formula (1) corresponds to the clinical finding that the instantaneous probability of recovery decreases as the duration of the major depressive episode lengthens [5].

From a pragmatic point of view, the finding that a cumulative probability distribution for durations within two years appears as an approximately straight line on double-logarithmic scales is important regardless of the exact model describing the distribution. We conducted an analysis of several cohort studies [1–5] and found that the survival curves of other cohort studies also appeared as an approximately linear line (data not shown). However, the lack of individual patient data prevented us from examining their fit to the models used in this review. To confirm the finding that the cumulative probability distribution appears as an approximately straight line on double-logarithmic scales, further reviews are necessary.

Mathematical models describing diseases are necessary not only for making predictions regarding the course of disease, but also for shedding light on the mechanisms of disease. Our finding will be helpful to developing a model describing the duration of major depressive disorder.

## Conclusion

Our findings showed that a log-normal distribution fit the distribution of major depressive episode durations better than that of an exponential distribution or a Weibull distribution. The distribution of the cumulative probabilities of major depressive disorder durations plotted on a double-logarithmic scale exhibited an approximately linear form. A log-normal model and a power law model may fit the distribution of major depressive episode durations.
